# A Porcelain Removable Bridge with Gum Pink Porcelain Saddle

**Published:** 1921-06

**Authors:** Joseph A. Carey


### A PORCELAIN REMOVABLE BRIDGE WITH GUM PINK PORCELAIN SADDLE

BY JOSEPH A. CAREY

Porcelain has always held a very prominent position in dental prosthesis, but not since the introduction of the porcelain jacket crown has so important an advancement in this most beautiful of all restorative materials taken place as is evidenced in the introduction of the Carey removable porcelain bridge with removable cast clasps.

The art of constructing gold removable bridgework has been materially developed and many ingenious devices have been provided to obtain almost ideal functioning dentures, yet up to the introduction of this bridge little or no progress has been made in the advance of porcelain bridgework in removable form.

Since modern dentistry has decreed that restorations that are movable are the nearest to a one-hundred-per-cent. prophylactic appliance, it was thought that porcelain had obtained its limit of usefulness, due mainly to three reasons: First, the inability to properly apply the attachments in such a manner that sufficient strength to resist an occlusal force be maintained; second, too bulky construction in the effort to overcome the first-named obstacle; third, it has been considered impossible to combine a porcelain bridge with cast gold clasps or other attachments of gold, the impression being, and rightly so, that the gold would melt in the process of fusing the porcelain, which requires nearly double the heat to fuse as is required of gold.

In this new work I have not only completely overcome all these obstacles or objections, but have evolved a bridge that combines unusual strength, and is even lighter and less bulky than the majority of gold removable work.

But the outstanding feature of this porcelain bridge is the esthetic appearance, with its hand-carved teeth and gum pink saddle or restoration of the alveolar tissue. This last-named feature is important. No restoration is complete unless all is restored; therefore, it may be rightly considered as a most complete restoration. This declaration may be countered by the statement that a vulcanite attachment will do all this. Let us see: Can vulcanite compare with the prophylactic qualities of porcelain? Will vulcanite take as kindly to the tissue with which it comes in contact? Will it compare, even to a small degree, in esthetic appearance? And, of course, the vulcanite attachment is much heavier and more bulky than porcelain.

This bridge is also designed so that, at the option of the operator, natural lateral motion of the abutment teeth may be maintained without further change in the construction of the work. Owing to the perfect fit of lug into the socket, this may be accomplished by leaving the lugs uncemented to the bridge. By simply cementing the clasps in position they may be held immovable.

This work is not confined to a continuous restoration, but may be applied equally as well to the connection of the right and left sides by means of lingual or palatine bar. This is especially valuable in restoration where there are no teeth posterior to the bridge, such as the replacement of the first, second and third molars. I have also succeeded in applying the Roach ball and socket attachment to this work. Here a slight change is necessitated in the bridge construction. Instead of the half-round tubing inserted in the bridge proper, a round tubing, the inside measurement of which is identical with the outside diameter of the extension protruding from the ball unit of the Roach attachment, is inserted. A thread is tapped in the tubing and one on the ball extension. This extension is simply screwed into position, and the result is a case that is equally as strong as gold.

I am now working on various other attachments and stress breakers, descriptions of which will be made public as soon as their application is assured.

### TECHNIQUE OF IMPRESSIONS

A most desirable and satisfying feature of removable bridgework having cast clasps, from the viewpoint of both the dentist and patient, is that no preparation or mutilation of abutment teeth is necessary. It is just a matter of taking impressions and completing of case. The technique of impression for the Carey removable porcelain bridge, although simple in detail, is none the less exacting. The complete set of impressions are four: First, the impressions for clasps. I would suggest a strict adherence to the Roach technique, the impressions being taken in complaster in a split or Roach tray; second, a split complaster impression of area to be restored with clasps in position after which they are removed from teeth and placed accurately in this impression; third, a modeling compound tray impression with clasps in position on abutments (clasps should not be transferred to this compound impression); fourth, a wax mash bite. In taking the bite it is suggested to take it in such a manner that just a mere imprint of the working side, or side that bridge is being made for, be taken, as this facilitates accuracy in articulating of model.

### DETAILS OF CONSTRUCTION

A model is made in artificial stone of the impression containing the clasps. A metal die is made of the impression devoid of clasps. A matrix of heavy-gauge platinum foil is swaged over metal model of saddle area, this transferred to the stone model and first bake applied to matrix on which is inserted in proper relation to each clasp a hollow, half-round, seamless ten-per-cent. iridio-platinum tubing of gauge sufficient to resist pressure of clasp lugs, thereby eliminating danger of fracture of porcelain. This tubing is the keystone of the bridge, as it absolutely protects bridge against fracture through stress of mastication. The tubing and lug I have made especially for this work and drawn out on especially made dies. It is not out of place to add here that the porcelain used in the first two bakes is also a specially made product, having a fusing point of 2,660 degrees Fahrenheit. I had this made for my previous work in fixed bridgework, the porcelain jacket crown bridge. It has found a place equally important in the removable work. In the third bake the bridge is carved to full occlusion and fused, and the fourth and last bake the gum pink porcelain is laid on the lingual and labial or buccal of saddle, festooned and fused.

The final operation, and a very difficult one, is the placing of the lugs or extension on the clasps in co-relation to the tube in the bridge proper. A hole is drilled through the plaster model to reach inside the clasp, at the approximal; the model is halved midway between the two abutments and rejoined with wax; a hole is drilled through the clasps at a point exactly opposite opening of the tube; lugs are inserted in position, waxed from the inside, carefully removed by drawing apart the halved model; lugs are soldered from the inside of clasps. This leaves the clasps perfectly flush at the bearing surface of the porcelain. Clasp, but not lug, is now polished and case is completed.

At the time the shade of teeth is taken, the shade of the gum tissue should also be noted, the reason of which is obvious. By carefully observing the technical details required in impression and construction of this work (and no detail is too small to be overlooked) this bridge will hold an important position in dental restoration, where beauty, strength and prophylactic value is combined.—*Dental Facts.*

